# Supplementary material for: The proteostatic landscape of healthy human oocytes
Source: EMBO J. 2025 Jul 16;44(16):4611–30. doi: 10.1038/s44318-025-00493-2 (PMC12361380; doi:10.1038/s44318-025-00493-2)
Supplement: Supplementary file 1 — Table EV1 [file 44318_2025_493_MOESM1_ESM.pdf]

Table EV1. Anonymized donors' information. Summary of the donors recruited for this study.
Proven fertility indicates whether the donor had experienced an unassisted pregnancy prior to the donation.
Donors shaded in grey were considered for aggregated quantifications only.

Donor n.	Ethnicity	Age	BMI	Proven fertility	GVs	MIIs	Color
1	Caucasian	33	25.9	Yes	2	2	
2	Caucasian	34	17.7	Yes	0	3	
3	Caucasian	32	24.9	No	2	1	
4	Caucasian	29	29.7	Yes	0	4	
5	Caucasian	24	19.8	No	0	5	
6	Caucasian	30	24.4	No	2	2	
7	Caucasian	29	22.7	Yes	0	8	
8	Caucasian	19	29.7	Yes	0	9	
9	Caucasian	26	21.5	Yes	1	3	
10	Caucasian	32	21.8	Yes	0	4	
11	Caucasian	29	22.8	No	0	5	
12	Caucasian	32	23.1	Yes	2	3	
13	Hispanic	28	21.5	No	7	7	
14	Caucasian	25	18.7	Yes	1	2	
15	Caucasian	34	25.9	Yes	1	3	
16	Caucasian	33	20	No	6	0	
17	Caucasian	31	24	No	5	0	
18	Caucasian	20	21.6	No	4	1	
19	Caucasian	33	27.6	No	0	4	
20	Caucasian	30	23.4	Yes	0	4	
21	Hispanic	28	32.1	No	0	3	
				**Total**	33	73	
