# Supplementary material for: The proteostatic landscape of healthy human oocytes
Source: EMBO J. 2025 Jul 16;44(16):4611–30. doi: 10.1038/s44318-025-00493-2 (PMC12361380; doi:10.1038/s44318-025-00493-2)
Supplement: Supplementary file 3 — Source data Fig. 1 [file 44318_2025_493_MOESM3_ESM.zip › Figure 1/A/Readme.rtf]

Raw Images shown in Fig. 1A. Images are split by channel. The channel and the oocyte type are indicated in the filename. Both single planes and MAX Z projections are shown.
