# Supplementary figures and images for: The proteostatic landscape of healthy human oocytes
Source: EMBO J. 2025 Jul 16;44(16):4611–30. doi: 10.1038/s44318-025-00493-2 (PMC12361380; doi:10.1038/s44318-025-00493-2)

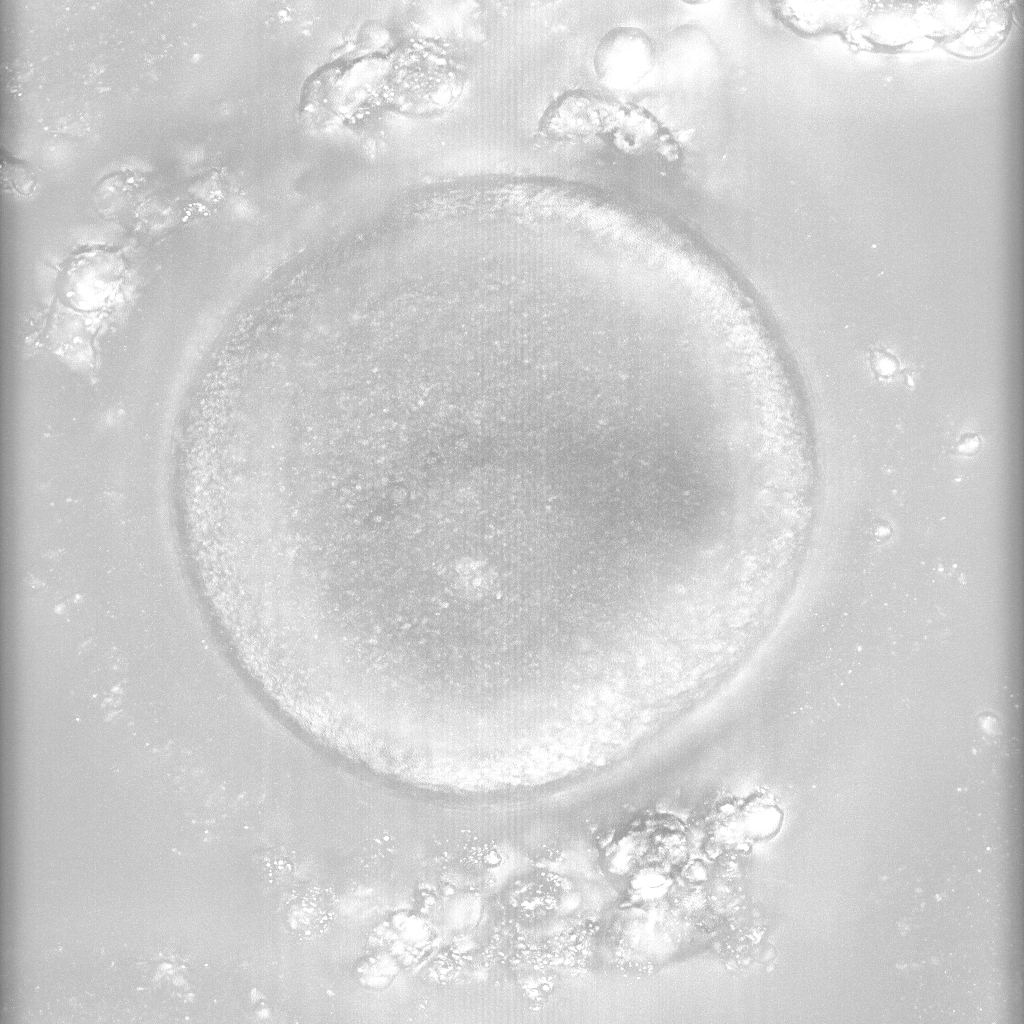

Supplement: Supplementary file 3 — Source data Fig. 1 [file 44318_2025_493_MOESM3_ESM.zip › Figure 1/A/MAX_GV_BF.tif]

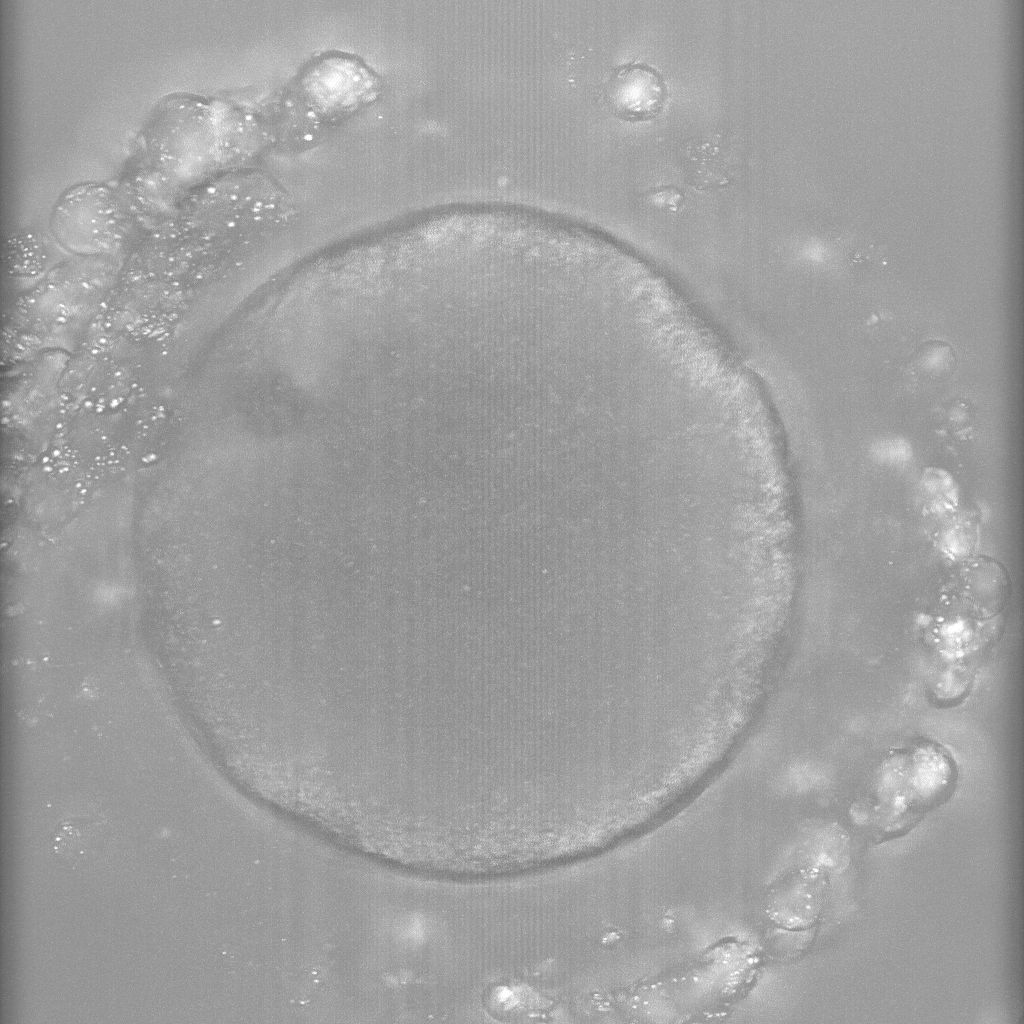

Supplement: Supplementary file 3 — Source data Fig. 1 [file 44318_2025_493_MOESM3_ESM.zip › Figure 1/A/MAX_MII_BF.tif]

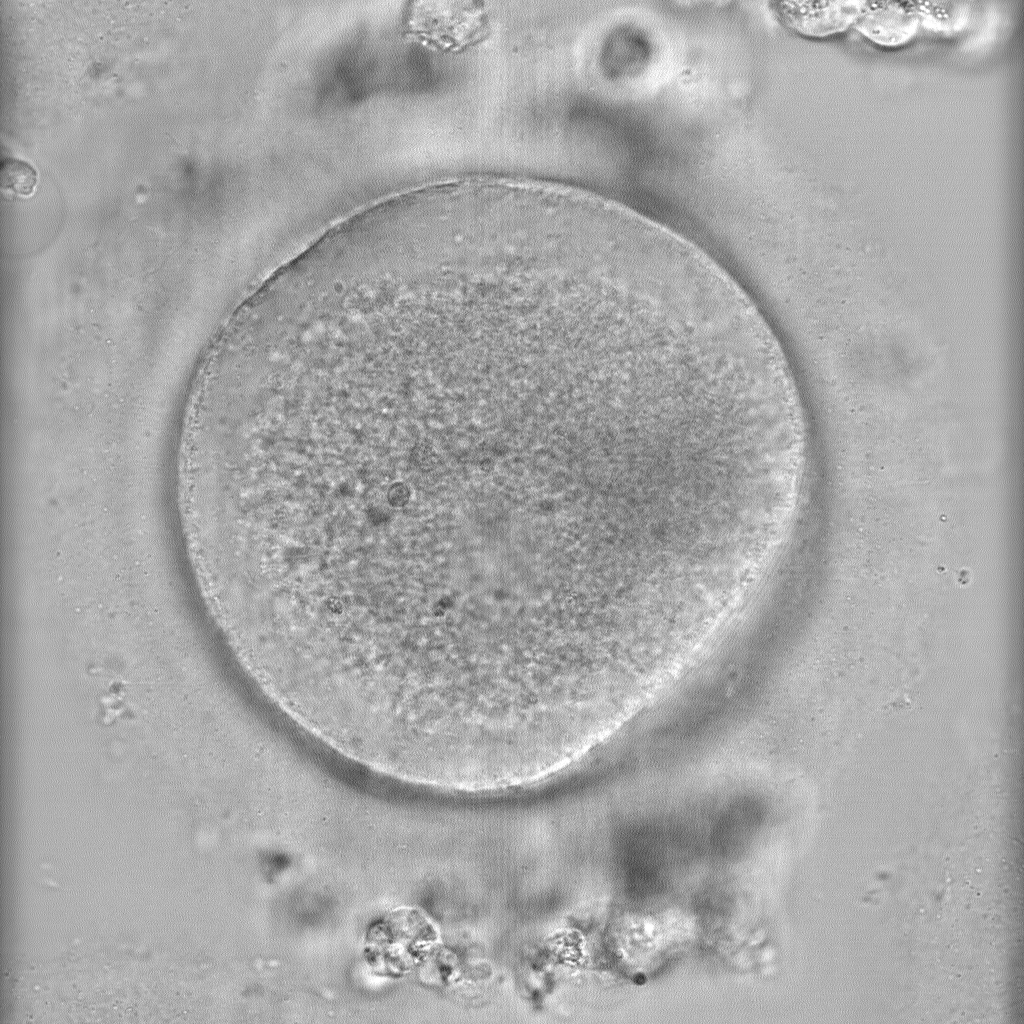

Supplement: Supplementary file 3 — Source data Fig. 1 [file 44318_2025_493_MOESM3_ESM.zip › Figure 1/A/GV_BF.tif]

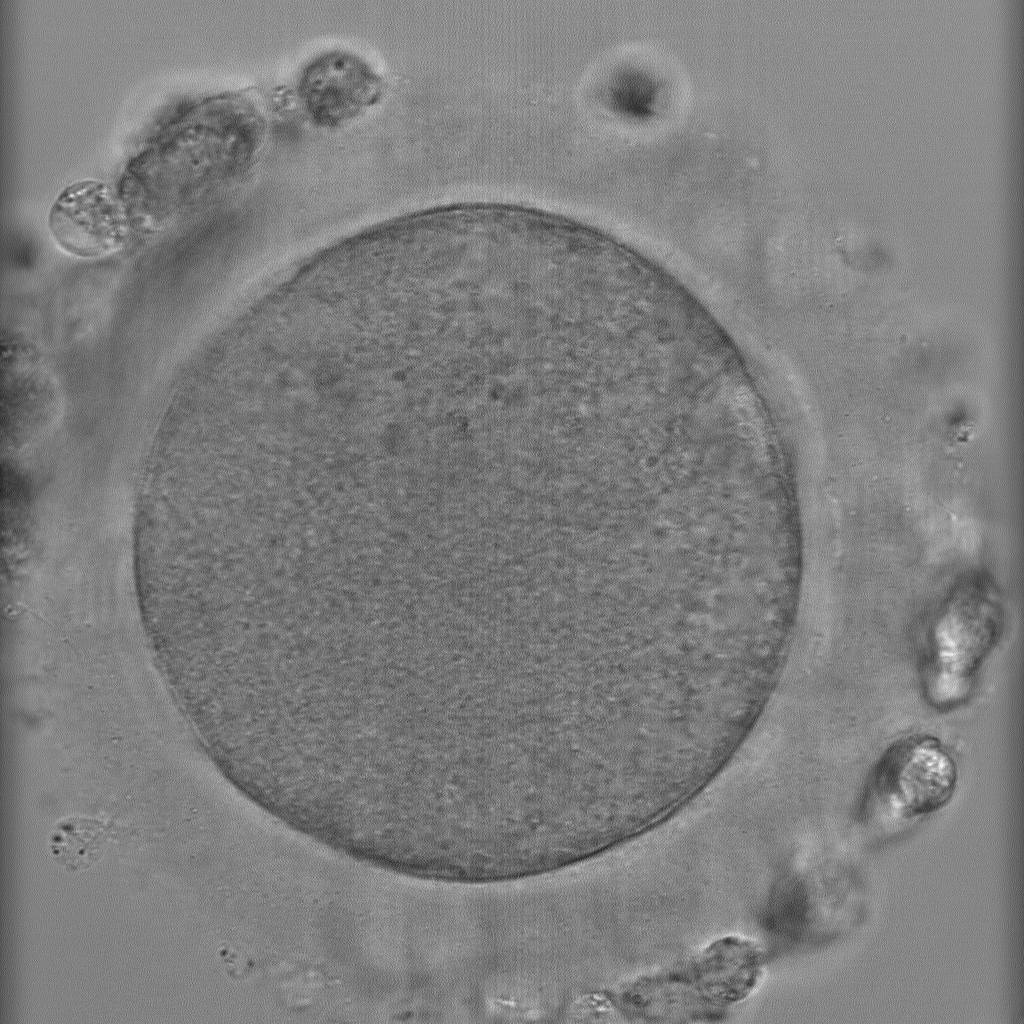

Supplement: Supplementary file 3 — Source data Fig. 1 [file 44318_2025_493_MOESM3_ESM.zip › Figure 1/A/MII_BF.tif]

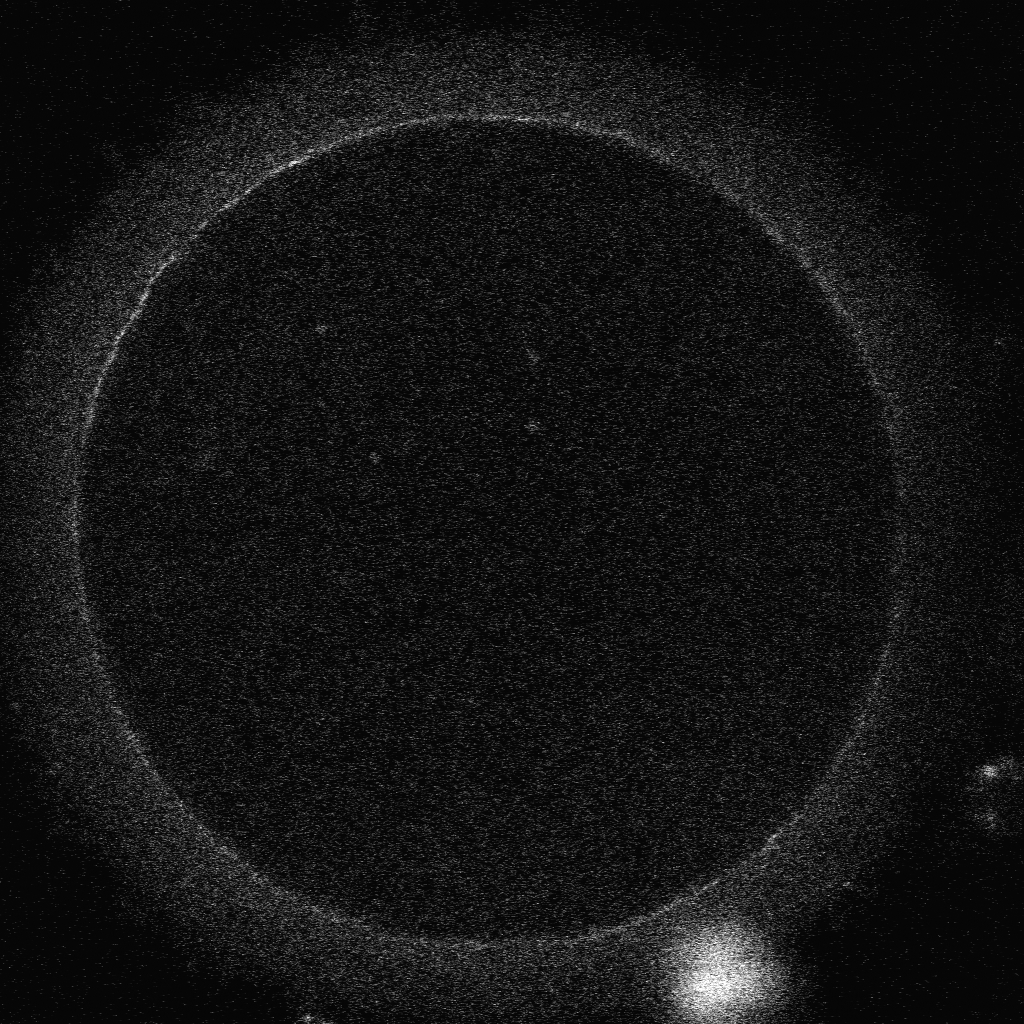

Supplement: Supplementary file 4 — Source data Fig. 2 [file 44318_2025_493_MOESM4_ESM.zip › Figure 2/G/MII.tif]

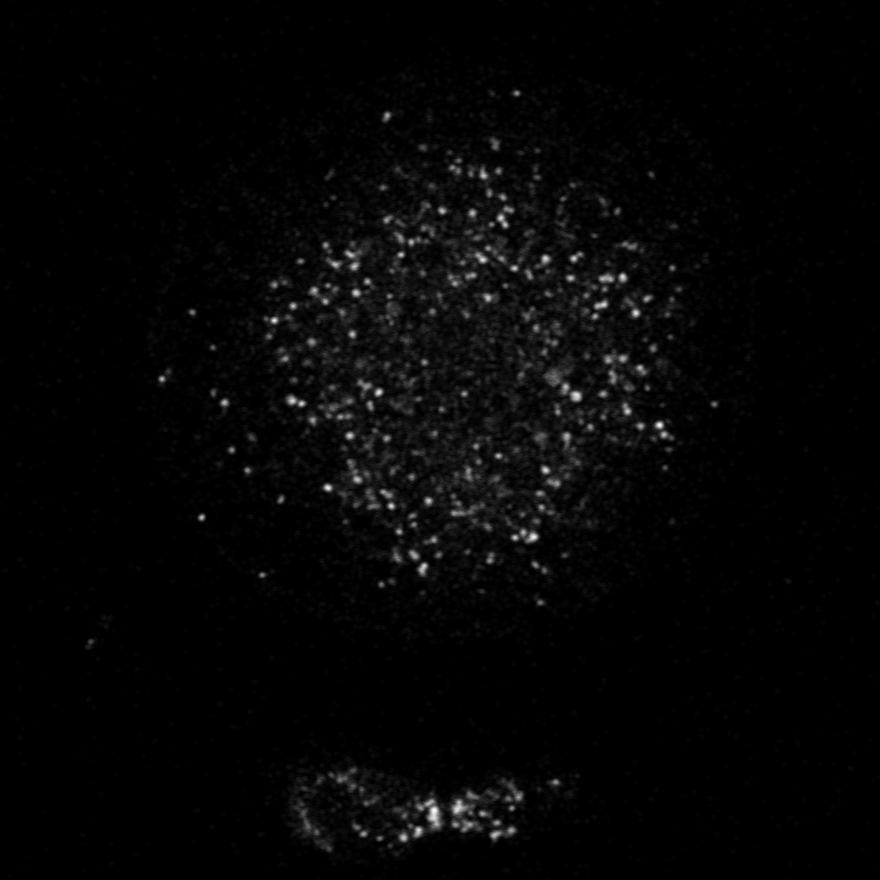

Supplement: Supplementary file 6 — Source data Fig. 4 [file 44318_2025_493_MOESM6_ESM.zip › Figure 4/A/GV.tif]

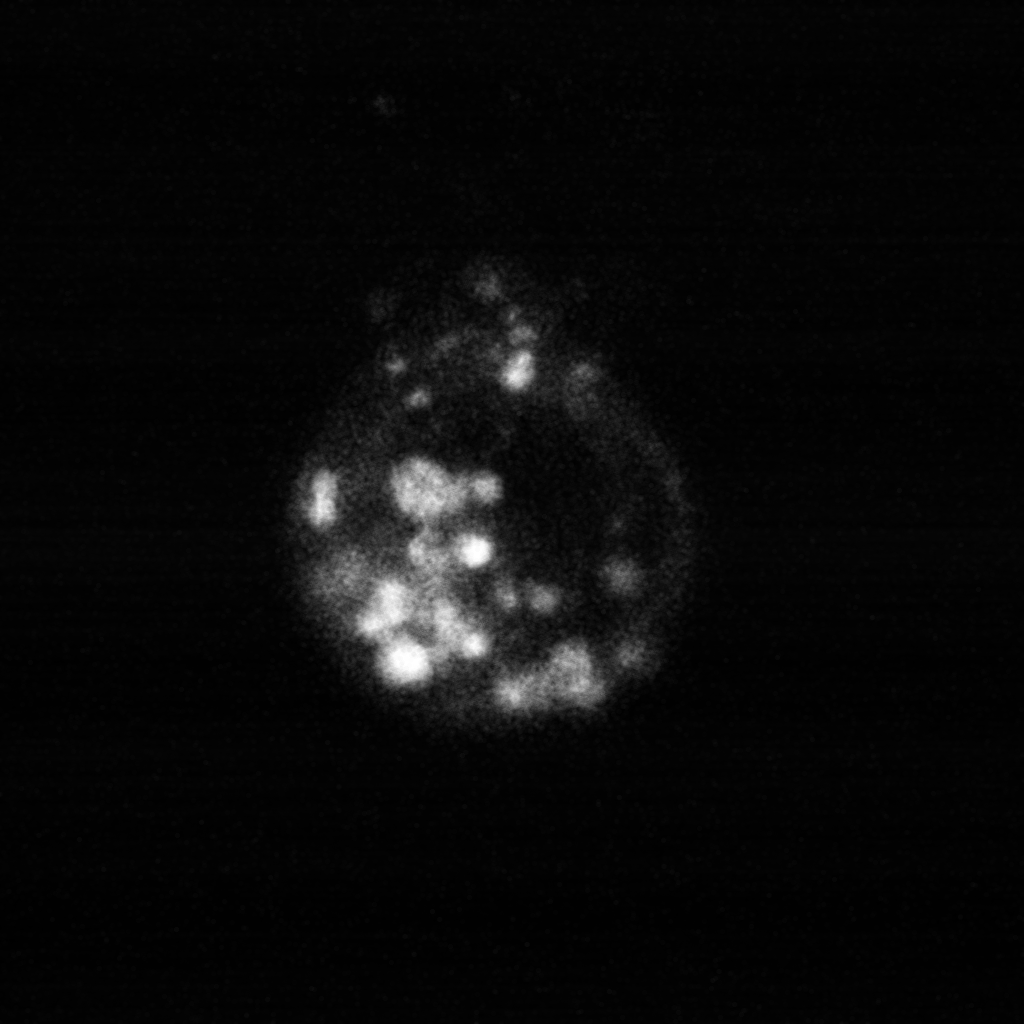

Supplement: Supplementary file 7 — Figure EV1-D Source Data [file 44318_2025_493_MOESM7_ESM.zip › Figure EV1/D/labelled.tif]
