# Supplementary material for: The proteostatic landscape of healthy human oocytes
Source: EMBO J. 2025 Jul 16;44(16):4611–30. doi: 10.1038/s44318-025-00493-2 (PMC12361380; doi:10.1038/s44318-025-00493-2)
Supplement: Supplementary file 4 — Source data Fig. 2 [file 44318_2025_493_MOESM4_ESM.zip › Figure 2/G/Readme.rtf]

Channel 1: LAMP1Channel 2: DNAChannel 3: BF
