# Supplementary material for: The proteostatic landscape of healthy human oocytes
Source: EMBO J. 2025 Jul 16;44(16):4611–30. doi: 10.1038/s44318-025-00493-2 (PMC12361380; doi:10.1038/s44318-025-00493-2)
Supplement: Supplementary file 8 — Expanded View Figures [file 44318_2025_493_MOESM8_ESM.pdf]

Expanded View Figures

Figure EV1. Morphometric analysis of the retrieved oocytes and validation of LysoTracker, TMRE and Me4Bpy labelling in human cumulus-oocyte complexes.

(**A**) Quantification of the oocyte diameter from live-cell imaging experiments. Data points represent individual oocytes and are colour-coded by donor (see Table EV1). The horizontal lines indicate the median of each distribution, boxes represent the interquartile ranges (IQR), and whiskers denote the minima and maxima, respectively. Numbers in parentheses indicate the total amount of oocytes quantified per condition from $N = 9$ and $N = 12$ donors, respectively. p value: unpaired t test with Welch's correction. (**B**) Representative confocal images of the chromatin configurations in GVs isolated from $N = 7$ donors and labelled with Hoechst 33342. Classification was performed according to Combelles et al (2002). The number of oocytes in each class is indicated. (**C**) Relative proportion of the chromatin configuration classes among the analysed GVs. The original data from Combelles et al (2002) are reported for comparison. Number into parentheses indicate the total amount of oocytes scored per study. The p value was calculated with a Fisher's exact test on the raw oocyte counts. (**D**) Representative live confocal images of Zona pellucida-attached cumulus cells unlabelled or labelled with LysoTracker Deep Red, Me4Bpy and TMRE to highlight active lysosomes, proteasomes, and mitochondria, respectively. (**E**) Representative live epifluorescence images of the same oocyte before and after CCCP treatment to dissipate the mitochondrial membrane potential. Mitochondria were labelled with the membrane potential-sensitive dye TMRE before CCCP treatment. (**F**) Live confocal images of GV-stage oocytes treated with or without Bafilomycin A1 and MG-132 to inhibit lysosomes and proteasomes, respectively. Oocytes were labelled in presence of Verapamil with LysoTracker, Me4Bpy and TMRE to highlight active lysosomes, proteasomes and mitochondria, respectively. DNA was counterstained with Hoechst 33342. (**G**) Representative live confocal images of Zona-attached cumulus cells treated with or without Bafilomycin A1 and MG-132 to inhibit lysosomes and proteasomes, respectively. Cells were labelled with LysoTracker Deep Red, Me4Bpy and TMRE to highlight active lysosomes, proteasomes and mitochondria, respectively. Notice that treated cells were still alive, as indicated by the retention of TMRE signal. Source data are available online for this figure.

 © The Author(s)

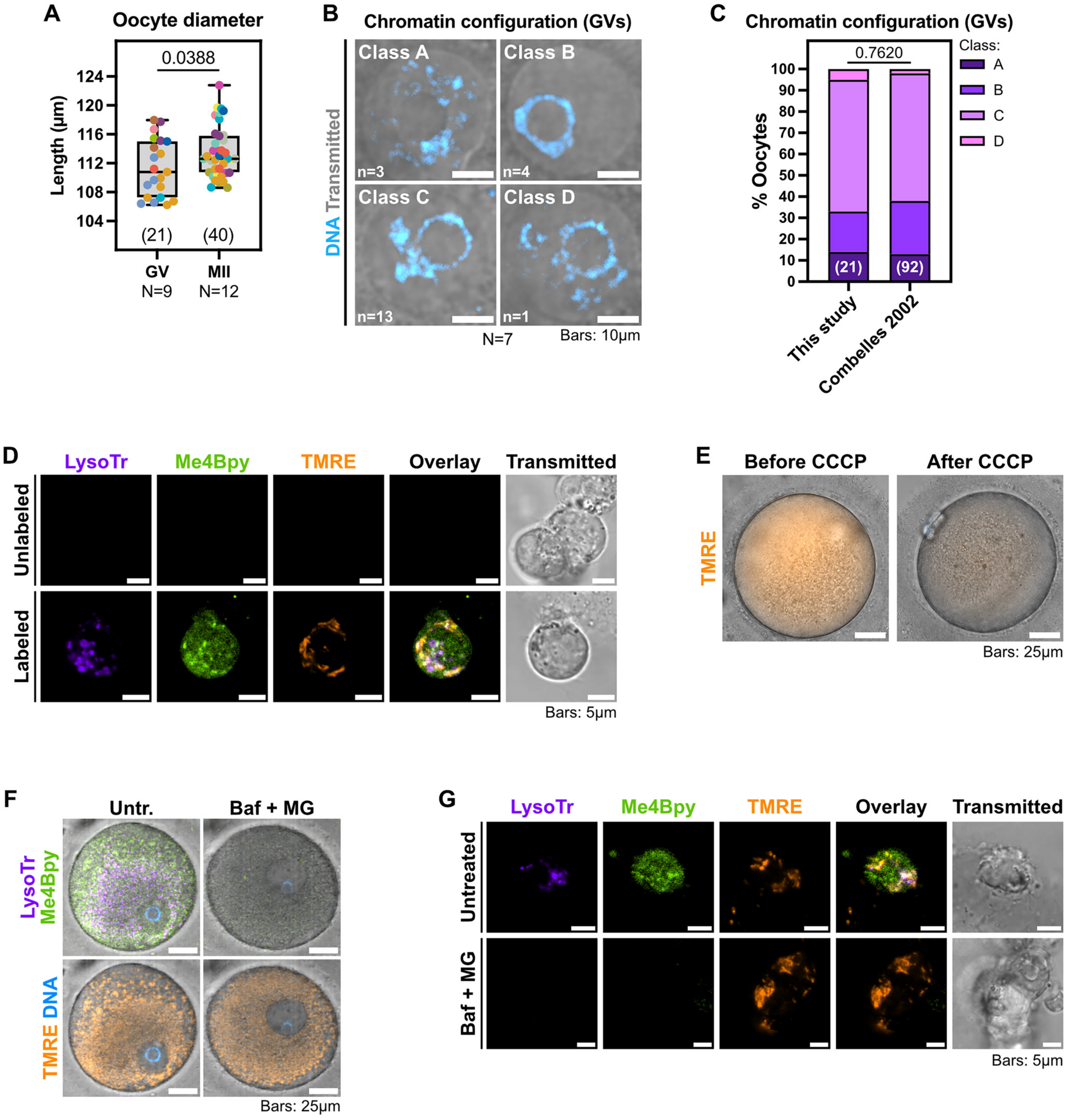
A
Oocyte diameter
0.0388
Length (μm)
124
120
116
112
108
104
(21)
(40)
GV
N=9
MII
N=12
B
Chromatin configuration (GVs)
DNA Transmitted
Class A
Class B
Class C
Class D
n=3
n=4
n=13
n=1
N=7
Bars: 10μm
C
Chromatin configuration (GVs)
0.7620
Class:
A
B
C
D
% Oocytes
(21)
(92)
This study
Combelles 2002
D
LysoTr
Me4Bpy
TMRE
Overlay
Transmitted
Unlabeled
Labeled
Bars: 5μm
E
Before CCCP
After CCCP
TMRE
Bars: 25μm
F
Untr.
Baf + MG
LysoTr Me4Bpy
TMRE DNA
Bars: 25μm
G
LysoTr
Me4Bpy
TMRE
Overlay
Transmitted
Untreated
Baf + MG
Bars: 5μm

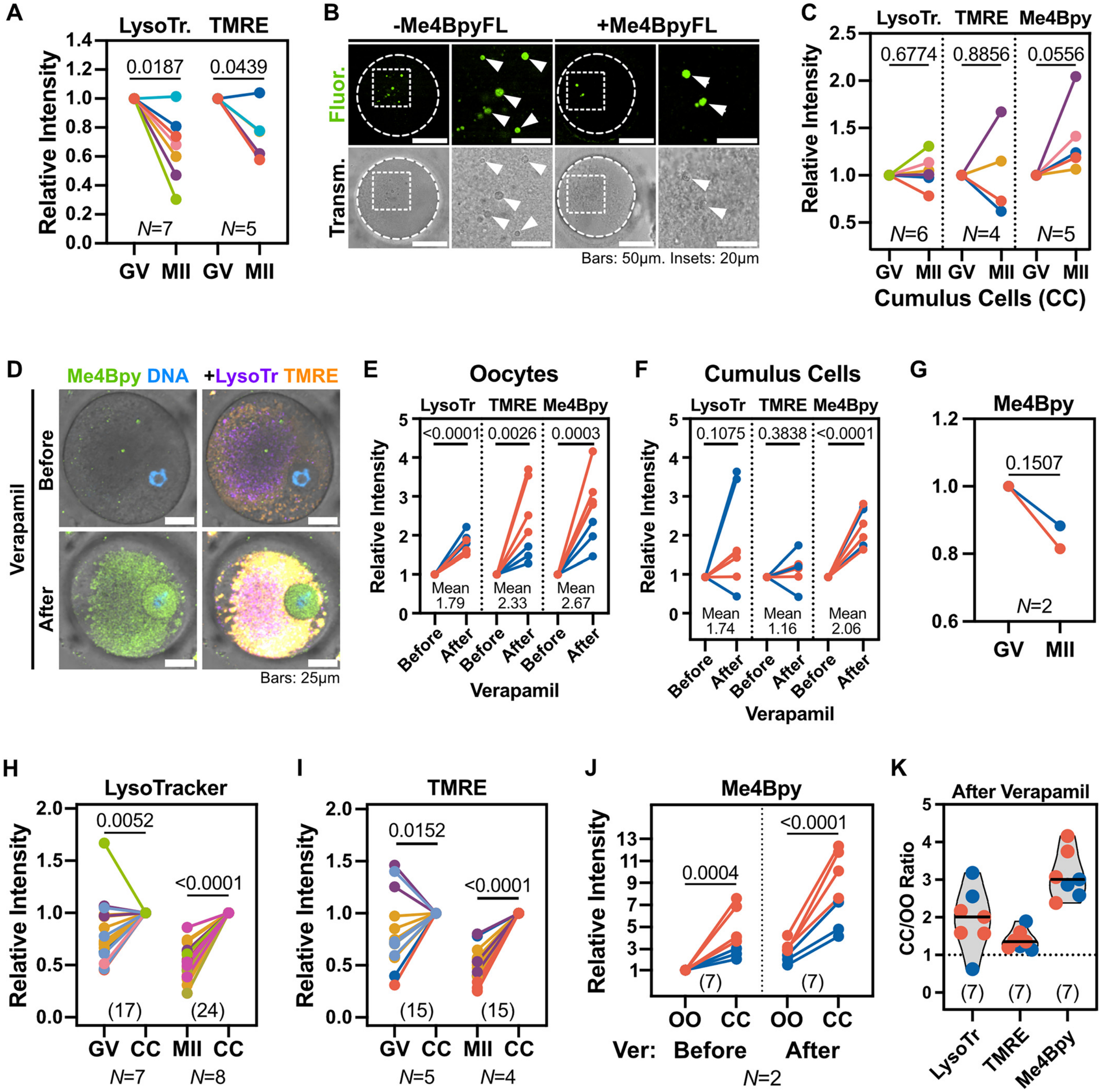
A
LysoTr.
TMRE
Relative Intensity
0.0187
0.0439
N=7
N=5
GV MII GV MII
B
-Me4BpyFL
+Me4BpyFL
Fluor.
Transm.
Bars: 50μm. Insets: 20μm
C
LysoTr. TMRE Me4Bpy
0.6774
0.8856
0.0556
N=6
N=4
N=5
Cumulus Cells (CC)
D
Me4Bpy DNA
+LysoTr TMRE
Verapamil
Before
After
Bars: 25μm
E
Oocytes
LysoTr TMRE Me4Bpy
<0.0001
0.0026
0.0003
Mean 1.79
Mean 2.33
Mean 2.67
Before After Before After Before After
Verapamil
F
Cumulus Cells
LysoTr TMRE Me4Bpy
0.1075
0.3838
<0.0001
Mean 1.74
Mean 1.16
Mean 2.06
Verapamil
G
Me4Bpy
0.1507
N=2
GV MII
H
LysoTracker
0.0052
<0.0001
(17)
(24)
GV CC MII CC
N=7
N=8
I
TMRE
0.0152
<0.0001
(15)
(15)
GV CC MII CC
N=5
N=4
J
Me4Bpy
0.0004
<0.0001
(7)
(7)
OO CC OO CC
Ver: Before After
N=2
K
After Verapamil
CC/OO Ratio
(7)
(7)
(7)
LysoTr
TMRE
Me4Bpy

 © The Author(s)

Figure EV2. Characterization of LysoTracker, TMRE, and Me4Bpy in oocytes and cumulus cells before and after Verapamil treatment.

(**A**) Comparison of the average Lysotracker (lysosomes) and TMRE (mitochondria) intensity in GV and MII oocytes as shown in Fig. 1B. Data from multiple oocytes were averaged by donor and normalized to the GV value for each donor. *N* numbers indicate the total donors considered for each quantification. *p* values: ratio-paired *t* tests. (**B**) Representative live confocal images of oocytes labelled with or without Me4Bpy. Arrowheads indicate autofluorescent refractile bodies. (**C**) Comparison of LysoTracker, TMRE and Me4Bpy intensity in cumulus cells attached to GV- and MII-stage oocytes from multiple donors. Data were averaged by donor and normalized, for each donor and dye, to the GV value. Only donors with at least one GV and one MII were considered. *p* values: ratio-paired *t* tests. (**D**) Representative live confocal images of the same oocytes labelled with LysoTracker, TMRE and Me4Bpy and imaged before and after incubation with Verapamil. DNA was counterstained with Hoechst 33342. (**E**, **F**) Quantification of LysoTracker, TMRE and Me4Bpy intensities in the same oocytes (**E**) and cumulus cells (**F**) before and after verapamil addition. Data points represent individual COCs from $N = 2$ donors. For each COC, data were normalized to the value before verapamil. *p* values: ratio-paired *t* tests. (**G**) Quantification of the average Me4Bpy intensity in Verapamil-treated GV and MII oocytes from $N = 2$ donors, as shown in Fig. 1D. Data from multiple oocytes were averaged by donor and normalized to the GV value for each donor. *p* value: ratio-paired *t* test. (**H**, **I**) Pairwise comparison of Lysotracker (**H**) and TMRE (**I**) intensities between oocytes and the corresponding cumulus cells (CC) as shown in Fig. 1E,F. Data points represent individual Cumulus Oocyte Complexes (COCs) and are colour-coded by donor. For each COC, data were normalized to the CC value. *p* values: ratio-paired *t* tests. (**J**) Pairwise quantification of Me4Bpy intensity in the same oocytes (OO) and cumulus cells (CC) before and after incubation with Verapamil (Ver) as shown in Fig. 1G. Data points represent individual COCs and were normalized to the OO value before Verapamil for each COC. *p* values: ratio-paired *t* tests. The comparison between oocyte intensities before and after Verapamil treatment is shown in Fig. EV2E. (**K**) Quantification of the LysoTracker, TMRE and Me4Bpy intensity ratio between each oocyte (OO) and its corresponding cumulus cells (CC) after verapamil addition.

© The Author(s)

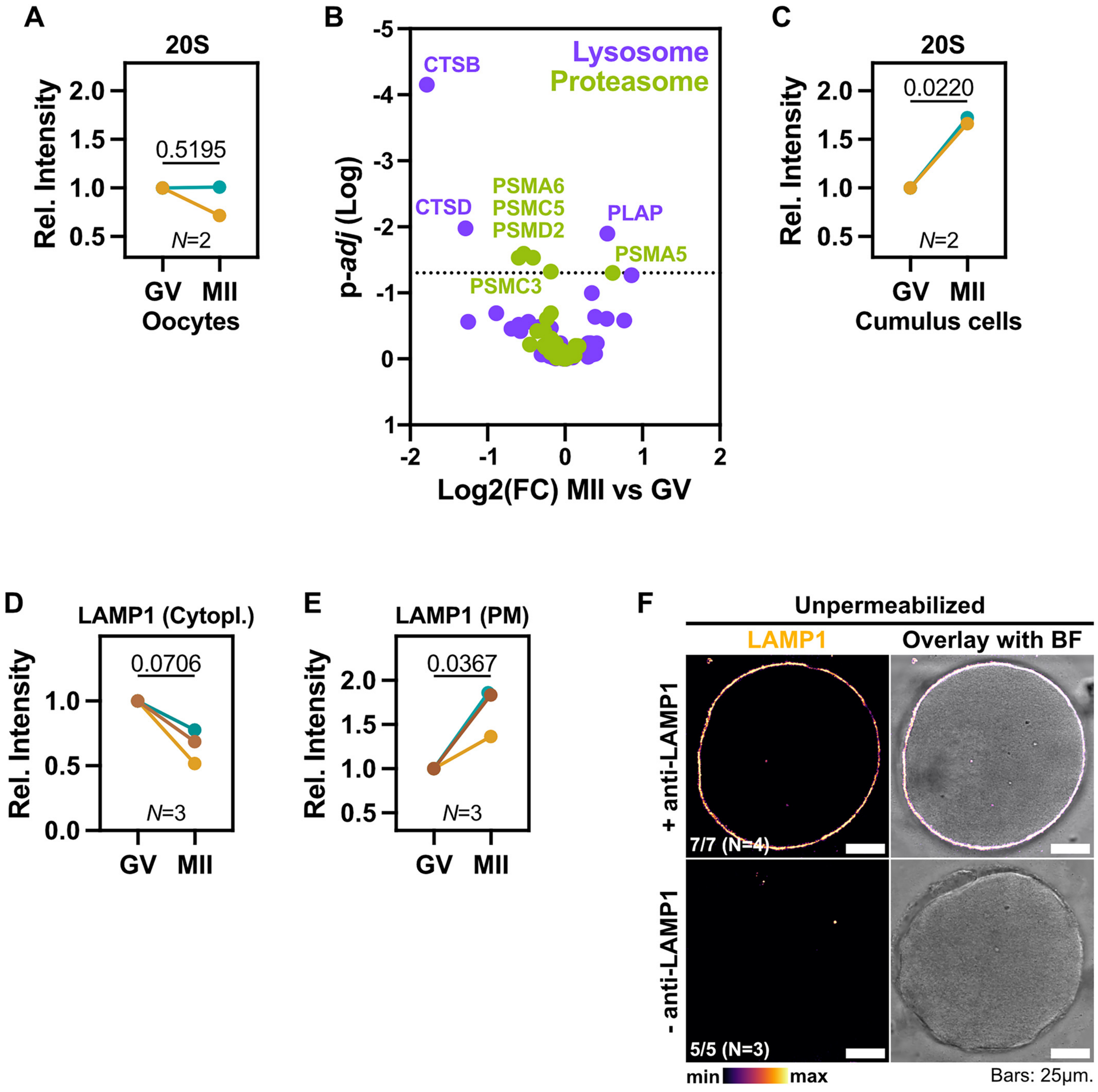

Figure EV3. The reduction in lysosome levels in MIIs is accompanied by lysosomal exocytosis.

(**A**, **C**) Comparison of the average 20S intensity in immunolabelled oocytes (**A**) and cumulus cells (**C**) from $N = 2$ donors. Data from multiple oocytes were averaged by donor and normalized to the GV value for each donor. *p* values: ratio-paired *t* tests. (**B**) Relative change in lysosomal (indigo) and proteasomal (green) proteins between GVs and MIIs, as detected by single-cell proteomics of human oocytes. Data were retrieved from (Galatidou et al, 2024). Proteins were selected based on GO Cellular Component (CC) terms matching "lysosome" and "proteasome", respectively. The dashed line indicates $p = 0.05$. (**D**, **E**) Quantification of the mean intracellular (**D**) or plasma membrane (**E**) LAMP1 intensity in GV and MII oocytes shown in Fig. 2D–F. For each donor ($N = 3$), data from multiple oocytes were averaged and normalized to the GV value. *p* values: ratio-paired *t* tests. (**F**) Representative confocal images of non-permeabilized MII oocytes labelled with or without anti-LAMP1 H3A4. LAMP1 intensity is displayed as a heatmap.

 © The Author(s)

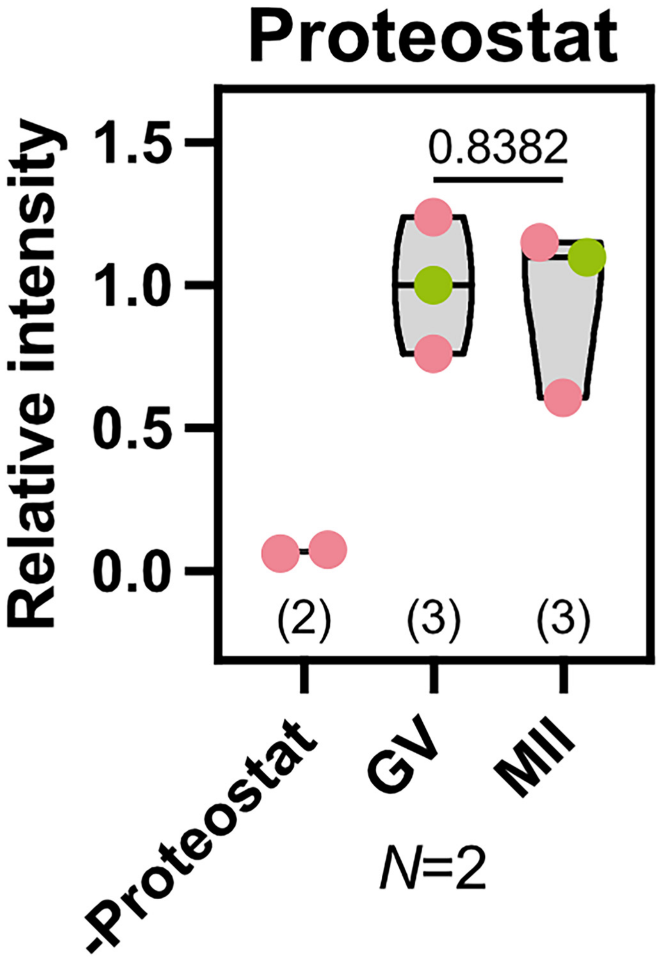

Figure EV4. Proteostat intensity does not change between GVs and MIIs.

Quantification of mean proteostat intensity in refractile bodies in GV and MII oocytes from $N = 2$ donors. Only donors with at least 1 GV and 1 MII were considered. Data points represent individual oocytes and were normalized to the median of GVs for each donor. Numbers in parentheses indicate the number of oocytes quantified per condition. *p* value: unpaired *t* test with Welch's correction.

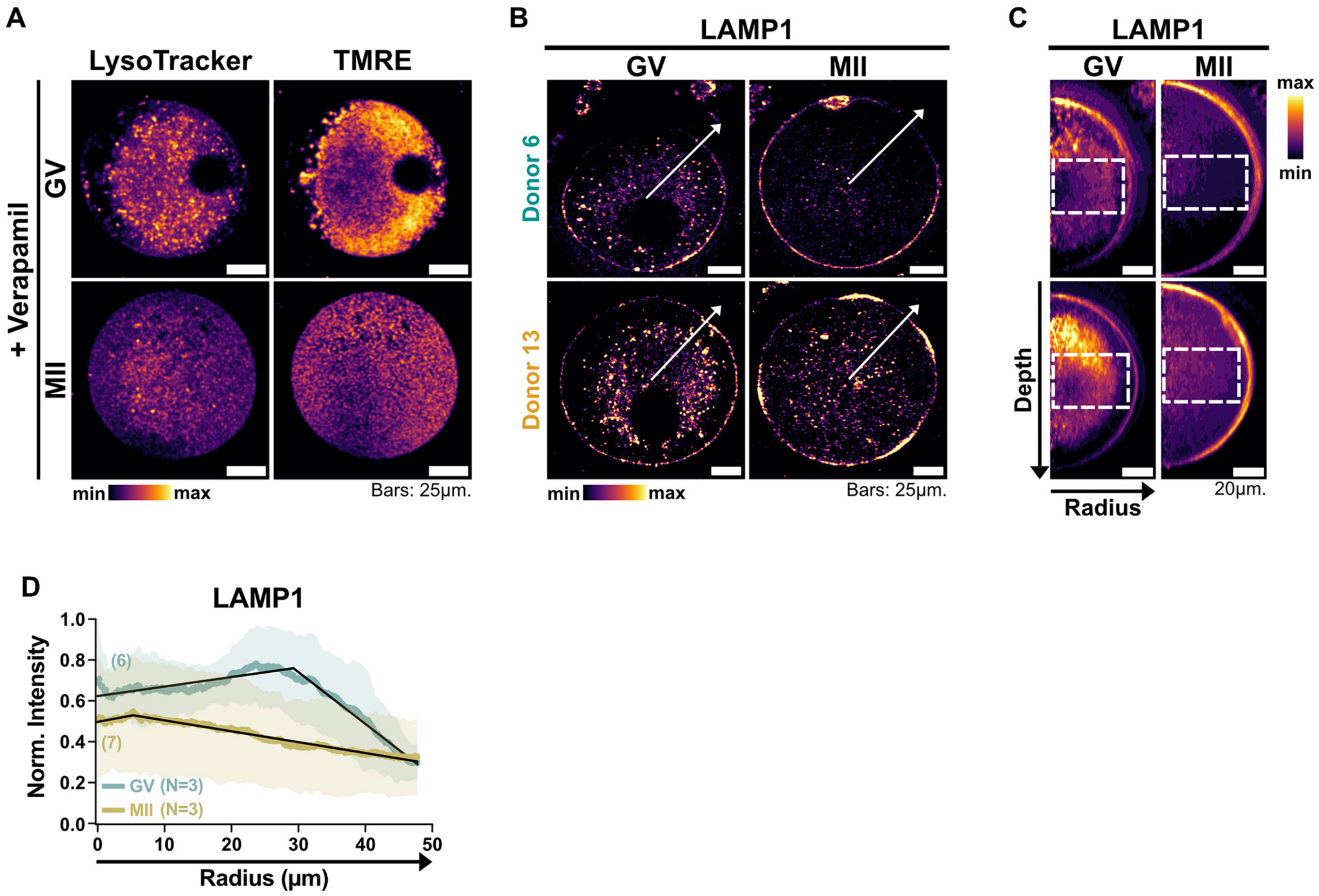

Figure EV5. Lysosomes are clustered in both human GVs and MIIs.

(**A**) Live confocal images of GV and MII oocytes labelled with LysoTracker and TMRE after Verapamil incubation. Intensities are displayed as heatmaps. Additional images of the same oocytes are shown in Fig. 4G. (**B**) Confocal images of GV and MII oocytes from multiple donors immunolabelled with anti-LAMP1 (shown as heatmap). Arrows indicate radiuses used for the radial reslicing shown in (**C**, **D**). Examples of oocytes from a third donor are shown in Fig. 2D. (**C**) Radial reslicing of the oocytes shown in (**B**) along the depicted radiuses. Average projections of 360 radiuses spaced by 1° are shown. Dashed boxes indicate the equatorial regions quantified in (**D**). (**D**) Radial distribution of LAMP1 signal in the cytoplasm of fixed oocytes. Mean and SD are shown. Numbers in parentheses indicate the total amount of oocytes quantified per condition from multiple donors. *N* indicates the number of donors considered per condition. Data were normalized to the min and max of each curve as 0 and 1, respectively. Note that the lower levels of LAMP1 antibody staining on MIIs cause a low signal-to-noise ratio, increasing the deviation on its average curve. Radius length does not include plasma membrane in these measurements. Black lines represent segmented models. Breakpoints of each model are as follows: GVs (29,260; 95% CI 28,836–29,684 μm), MIIs (5308 μm; 95% CI 4573–6043 μm).

 © The Author(s)
